# Association between body mass index and presence of carotid plaque among low-income adults aged 45 years and older: a population-based cross-sectional study in rural China

**DOI:** 10.18632/oncotarget.17608

**Published:** 2017-05-04

**Authors:** Yongzhong Lou, Bin Li, Lan Su, Zhenhong Mu, Minghao Sun, Hongfei Gu, Jingxian Ni, Yanan Wu, Jun Tu, Jinghua Wang, Xianjia Ning

**Affiliations:** ^1^ Department of Neurology, Tianjin Haibin People's Hospital, Tianjin, China; ^2^ Department of Neurology, Tianjin Medical University General Hospital, Tianjin, China; ^3^ Department of Epidemiology, Tianjin Neurological Institute, Tianjin, China; ^4^ Tianjin Neurological Institute, Key Laboratory of Post-Neuroinjury Neuro-Repair and Regeneration in Central Nervous System, Ministry of Education and Tianjin City, Tianjin, China; ^5^ Center of Clinical Epidemiology, Tianjin Medical University General Hospital, Tianjin, China

**Keywords:** carotid plaque, body mass index, ultrasonography, low-income population, epidemiology

## Abstract

Carotid plaque is a good surrogate endpoint for assessing arterial atherosclerosis, and atherosclerosis is a reliable predictor of cardiovascular diseases. However, the effect of body mass index on carotid plaque is unknown. Therefore, we aimed to explore the association between body mass index and carotid plaque in a low-income Chinese population. Residents aged ≥45 years and free of stroke and cardiovascular diseases were enrolled and divided into four groups based on body mass index. B-mode ultrasonography was performed to measure carotid plaque. The mean age of participants was 59.92 years overall. Significant correlations were observed between the presence of carotid plaque and male sex, older age, systolic blood pressure, fasting plasma glucose, and low-density lipoprotein cholesterol among the different BMI subgroups. Male sex increased the risk of carotid plaque in the overweight and obese groups. Older age and high level of low-density lipoprotein cholesterol were the independent risk factor for carotid plaque in four groups. Increased systolic blood pressure was an independent risk factor in the normal-weight, overweight, and obese groups; however, fasting plasma glucose was only significant in the normal-weight group. Thus, controlling the levels of low-density lipoprotein cholesterol, systolic blood pressure, and fasting plasma glucose is required to reduce carotid plaque risk.

## INTRODUCTION

The World Health Organization stated that 17.5 million people died of cardiovascular diseases (CVDs) in 2012, comprising 31% of all deaths globally. An estimated 7.4 million of those were due to coronary heart disease, and 6.7 million were due to stroke [1]. Of the 16 million deaths of individuals < 70 years of age due to non-communicable diseases, 82% occurred in low- and middle-income countries, and 37% were caused by CVDs [1]. CVDs are the leading cause of death both in Western countries and in China, accounting for 40% of deaths in China in 2012 [2]. Among those, 44.6% of deaths in rural areas and 42.5% in urban areas were CVD-related [2]. Stroke is the second most common cause of death; Feigin et al. reported that the age-standardized stroke rate in low- and middle-income countries is 1.24-fold higher than that in high-income countries [3]. Furthermore, the stroke prevalence among patients ≤75 years in low- and middle-income countries is three times higher than that in high-income countries [3]. Thus, individuals with CVD, or who are at high risk for it, require early detection and management to prevent or delay end events.

Several studies have shown that atherosclerosis is a reliable predictor for CVDs and that that presence of carotid plaque is a better surrogate endpoint for assessing arterial atherosclerosis lesions than is carotid intima-media thickness [4–9]. Several studies have also investigated risk factors for carotid plaque, such as age, sex, cigarette smoking, alcohol consumption, body mass index (BMI), education levels, and so on [6, 9–20]. However, there has been no consensus on the effect of BMI on carotid plaque. A study from Iceland showed that BMI protected against carotid plaque. Sturlaugsdottir et al. [9] demonstrated that after adjustment for other confounding factors, the risk of developing carotid plaque decreased by 28% for each 5-unit increase in BMI (odds ratio [OR], 0.72; 95% confidence interval [CI], 0.64-0.82; *P* < 0.001). In contrast to those results, West et al. [15] reported that BMI appeared to be a risk factor for carotid plaque among 2,448 young Finnish adults. They studied the risk of carotid atherosclerotic plaque in their adulthood following exposure to parental smoking in childhood and found that the average BMI in individuals without carotid plaque was 17.8 kg/m^2^, while that in individuals with carotid plaque was 19.1 kg/m^2^. Furthermore, several studies have demonstrated that BMI had no association with carotid plaque [8, 19, 21]. There are even fewer studies in Chinese populations. Because of the genetic differences between Chinese and Caucasian populations, conclusions from Caucasian populations regarding the associations between BMI and carotid plaque cannot be generalized to Chinese populations. Thus, the purpose of the present population-based study was to determine the association between BMI and carotid plaque among a low-income population in rural China.

## RESULTS

### Demographic characteristics for subjects

**Figure 1:**
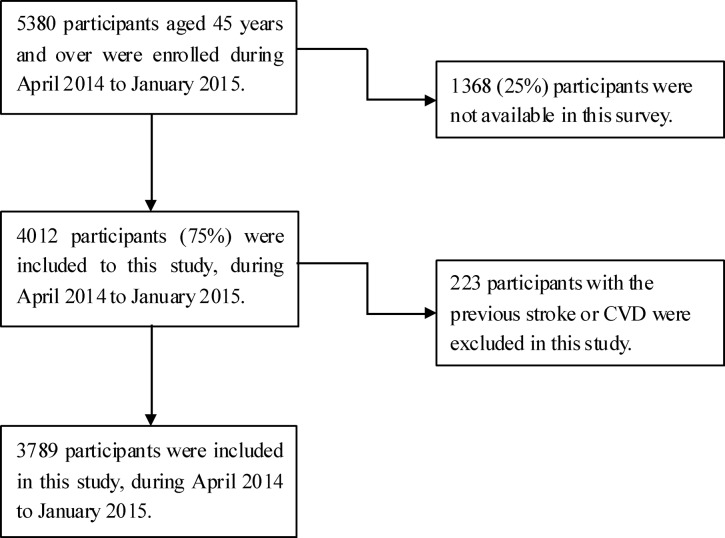
Flow chart of participants selection

In the present study, 4,012 participants were interviewed from among 5,380 eligible residents aged ≥45 years during the study period. The response rate was 74.5%. Finally, 3,789 subjects were included in our study, after excluding 223 residents with a previous history of CVD or stroke (Figure 1).

Among the 3,789 subjects, there were 1,560 men (41.2%) and 2,229 women (58.8%). The average age was 59.92 (9.70) years overall, 61.13 (9.90) years for men, and 59.07 (9.47) years for women (*P* < 0.001). The mean length of formal education was 5.48 years, and 17.4% of participants had never received a formal education (8.8% of men and 23.4% of women); 37.9% had gained a formal education ≥6 years. Men were more likely to have higher educational attainment, to be cigarette smokers, to consume alcohol, and to have hypertension; however, women had higher rates of illiteracy and obesity. Systolic blood pressure (SBP) and diastolic blood pressure (DBP) were higher in this population, with mean values of 146.42 mmHg and 86.81 mmHg, respectively. Moreover, SBP and DBP were higher in men than in women, but BMI, total cholesterol (TC), triglycerides (TG), high-density lipoprotein cholesterol (HDL-C), and low-density lipoprotein cholesterol (LDL-C) were greater in women than in men (Table 1).

**Table 1:** The demographical characteristic and risk factors in this population by gender

Risk factors	Total	Men	Women	*P*
Total:	3789 (100)	1560 (41.2)	2229 (58.8)	
Age, means (SD), years	59.92 (9.70)	61.13 (9.90)	59.07 (9.47)	<0.001
Age group, n (%)				<0.001
45~54 years	1236 (32.6)	430 (27.6)	806 (35.2)	
55~64 years	1514 (40.0)	632 (40.5)	882 (39.6)	
65~74 years	724 (19.1)	338 (21.7)	386 (17.3)	
≥75 years	315 (8.3)	160 (10.3)	155 (7.0)	
Education, means (SD), years	5.48 (6.54)	6.40 (3.22)	4.84 (3.61)	<0.001
Education, n (%)				<0.001
0 years	659 (17.4)	137 (8.8)	522 (23.4)	
1~6 years	1694 (44.7)	699 (44.8)	995 (44.6)	
> 6 years	1436 (37.9)	724 (46.4)	712 (31.9)	
Smoking status, n (%)				<0.001
Never smoking	2840 (75.0)	664 (42.6)	2176 (97.6)	
Smoking	949 (25.0)	896 (57.4)	53 (2.4)	
Alcohol consumption, n (%)				<0.001
Never drinking	3198 (84.4)	999 (64.0)	2199 (98.7)	
Drinking	591 (15.6)	561 (36.0)	30 (1.3)	
BMI				<0.001
Underweight	66 (1.7)	27 (1.7)	39 (1.7)	
Normal	1232 (32.5)	557 (35.7)	675 (30.3)	
Overweight	1603 (42.3)	653 (41.9)	950 (42.6)	
Obesity	888 (23.4)	323 (20.7)	565 (25.3)	
Hypertension, n (%)	2583 (68.2)	1111 (71.2)	1472 (66.0)	0.001
Diabetes, n (%)	533 (14.1)	216 (14.1)	317 (14.5)	0.719
Central obesity, n (%)	2317 (61.3)	778 (50.0)	1539 (69.2)	0.001
SBP, means (SD), mmHg	146.42 (22.17)	147.76 (21.41)	145.49 (22.64)	0.002
DBP, means (SD), mmHg	86.81 (11.40)	88.50 (11.22)	85.62 (11.39)	<0.001
BMI, means (SD), Kg/m^2^	25.57 (3.68)	25.20 (3.44)	25.82 (3.82)	<0.001
FPG, means (SD), mmol/L	5.92 (1.57)	5.91 (1.42)	5.93 (1.67)	0.660
TC, means (SD), mmol/L	4.87 (1.09)	4.62 (1.00)	5.04 (1.11)	<0.001
TG, means (SD), mmol/L	1.76 (1.24)	1.61 (1.24)	1.87 (1.22)	<0.001
HDL-C, means (SD), mmol/L	1.46 (0.46)	1.39 (0.43)	1.50 (0.48)	<0.001
LDL-C, means (SD), mmol/L	2.70 (1.25)	2.61 (1.20)	2.76 (1.28)	<0.001
LDL-C/HDL-C ratio	2.02 (1.22)	2.05 (1.32)	1.99 (1.14)	0.103

SD, standard deviation; SBP, systolic blood pressure; DBP, diastolic blood pressure; BMI, body mass index; FPG, fasting plasma glucose; TC, total cholesterol; TG, triglycerides; HDL-C, high density lipoprotein cholesterol; LDL-C, low density lipoprotein cholesterol.

### Prevalence of carotid plaque by demographic characteristics and risk factors

Table 2 shows the prevalence of carotid plaque by demographic characteristics and risk factors for all participants in this study. There was a significantly higher prevalence of carotid plaque associated with male sex, older age, cigarette smoking, hypertension, and diabetes mellitus (DM) (all *P* < 0.001); the plaque prevalence descreased with increasing BMI (*P* = 0.008); meanwhile the prevalence of plaque decreased with advancing education level (*P* < 0.001).

**Table 2:** The prevalence of carotid plaque by Demographical Characteristics and risk factors for all participants in this study

Characteristics/Risk factors	n (%)	Statistic	*P*
Gender:	1574 (41.5)	80.516	<0.001
Men	782 (50.1)		
Women	792 (35.5)		
Age group:		335.127	<0.001
45~54 years	281 (22.7)		
55~64 years	684 (45.2)		
65~74 years	390 (53.9)		
≥75 years	219 (69.5)		
Education group:		48.071	<0.001
0 year	318 (48.3)		
1 year∼	759 (44.8)		
≥6 years	497 (34.6)		
Risk factors:			
Smoking status:		32.367	<0.001
Never smoking	1105 (38.9)		
Smoking	469 (49.4)		
Alcohol consumption:		2.526	0.112
Never drinking	1311 (41.0)		
Drinking	263 (44.5)		
Hypertension:		98.154	<0.001
Yes	1213 (47.0)		
No	361 (29.9)		
Diabetes:		33.486	<0.001
Yes	282 (52.9)		
No	1263 (39.6)		
BMI:		11.964	0.008
Underweight	38 (57.6)		
Normal	539 (43.8)		
Overweight	646 (40.3)		
Obesity	351 (39.5)		
Central obesity		1.282	0.258
Yes	946 (60.2)		
No	1371 (62.0)		

### Differences in risk factors based on the presence or absence of carotid plaque

There was a significant discrepancy in mean BMI, SBP, DBP, fasting plasma glucose (FPG), TC, LDL-C, and the LDL-C/HDL-C ratio between individuals with or without carotid plaque (all *P* < 0.05). However, TG, HDL-C, and waist circumference were similar between individuals with or without carotid plaque (all *P* > 0.05) (Table 3).

**Table 3:** The differences in measurements of risk factors between presence and absence of carotid plaque in this population

Measurement	Presence carotid plaque	Absence carotid plaque	*P*
BMI, Kg/m^2^,	25.35 (3.70)	25.72 (3.67)	0.002
SBP, mmHg	151.58 (23.25)	142.76 (20.60)	<0.001
DBP, mmHg	87.32 (11.56)	86.45 (11.28)	0.021
FPG, mmol/L	6.09 (1.83)	5.81 (1.34)	<0.001
TC, mmol/L	4.99 (1.15)	4.78 (1.04)	<0.001
TG, mmol/L	1.76 (1.13)	1.76 (1.31)	0.901
HDL-C, mmol/L	1.45 (0.45)	1.46 (0.47)	0.582
LDL-C, mmol/L	3.07 (1.44)	2.43 (1.02)	<0.001
LDL-C/HDL-C	2.33 (1.54)	1.79 (0.87)	<0.001
Waist circumference, cm	25.35 (3.70)	89.11 (9.11)	0.199

SBP, systolic blood pressure; DBP, diastolic blood pressure; BMI, body mass index; FPG, fasting plasma glucose; TC, total cholesterol; TG, triglycerides; HDL-C, high density lipoprotein cholesterol; LDL-C, low density lipoprotein cholesterol.

### The prevalence of carotid plaque in the stratified analysis

Tables 4 and 5 show the prevalence of carotid plaque by BMI for all participants in this study. The prevalence of carotid plaque was higher for men and older individuals among all four weight groups (underweight, normal-weight, overweight, and obese; all *P* < 0.05). Carotid plaque prevalence decreased with advancing education level for all groups except the underweight group (*P* = 0.090). Moreover, the prevalence of carotid plaque was higher in patients with hypertension and DM and among cigarette smokers in the normal-weight, overweight, and obese groups (all *P* < 0.05).

**Table 4:** The prevalence of carotid plaque by BMI for all participants in this study

Characteristics/Risk factors	Underweight		Normal		Overweight		Obesity	
	n (%)	*P*	n (%)	*P*	n (%)	*P*	n (%)	*P*
Gender:		0.011		< 0.001		< 0.001		0.002
Men	21 (77.8)		280 (50.3)		332 (50.8)		149 (46.1)	
Women	17 (43.6)		259 (38.4)		314 (33.1)		202 (35.8)	
Age group:		0.013		< 0.001		< 0.001		< 0.001
45~54 years	2 (18.2)		81 (22.6)		112 (21.3)		86 (25.4)	
55~64 years	14 (60.9)		218 (45.8)		283 (44.0)		169 (45.4)	
65~74 years	15 (78.9)		140 (55.3)		163 (51.9)		52.2 (72)	
≥75 years	7 (53.8)		100 (69.4)		88 (73.9)		24 (61.5)	
Education group:		0.090		< 0.001		0.001		0.008
0 year	15 (62.5)		118 (52.4)		123 (46.6)		62 (42.5)	
1 year∼	17 (68.0)		267 (46.9)		300 (42.7)		175 (44.0)	
≥6 years	6 (35.3)		154 (35.2)		223 (35.0)		114 (33.1)	
Risk factors:								
Hypertension		0.805		< 0.001		< 0.001		< 0.001
Yes	18 (60.0)		373 (50.3)		512 (46.3)		310 (43.8)	
No	20 (55.6)		166 (33.8)		134 (26.9)		41 (22.7)	
Diabetes				< 0.001		< 0.001		0.020
Yes	−		68 (61.3)		132 (53.7)		82 (46.6)	
No	38 (58.5)		462 (42.0)		498 (37.6)		265 (37.7)	
Current smoking		0.090		0.001		< 0.001		0.044
Yes	12 (75.0)		173 (50.7)		199 (49.3)		85 (45.2)	
No	26 (52.0)		366 (41.1)		447 (37.3)		266 (38.0)	
Alcohol drinking		0.451		0.646		0.410		0.179
Yes	6 (75.0)		94 (45.2)		112 (42.6)		51 (45.5)	
No	32 (55.2)		445 (43.5)		534 (39.9)		300 (38.7)	
Central obesity		0.397		0.055		0.864		0.169
Yes	−		137 (48.9)		469 (40.2)		340 (39.2)	
No	35 (61.4)		402 (42.2)		177 (40.7)		11 (55.0)	

**Table 5:** The mean value of measurements in groups with or without carotid plaque by BMI group

Risk factors	Underweight		Normal		Overweight		Obesity	
	Means (SD)	*P*	Means (SD)	*P*	Means (SD)	*P*	Means (SD)	*P*
Waist circumference, cm		0.608		<0.001		0.001		0.169
CP	73.800		82.627		90.683		99.433	
No-CP	72.740		81.300		89.735		98.790	
SBP, mmHg		0.509		<0.001		<0.001		<0.001
CP	138.293		149.229		152.650		154.669	
No-CP	134.101		139.174		142.652		148.018	
DBP, mmHg		0.959		0.139		0.001		0.885
CP	78.272		84.400		88.424		90.739	
No-CP	78.435		83.484		86.481		90.628	
TC, mmol/L		0.143		<0.001		0.003		0.125
CP	4.923		4.972		4.962		5.075	
No-CP	4.636		4.614		4.802		4.953	
TG, mmol/L		0.971		0.241		0.355		0.312
CP	1.192		1.439		1.870		2.099	
No-CP	1.196		1.380		1.810		2.195	
HDL-C, mmol/L		0.878		0.801		0.022		0.575
CP	1.840		1.588		1.379		1.331	
No-CP	1.859		1.594		1.433		1.314	
LDL-C, mmol/L		0.002		<0.001		<0.001		<0.001
CP	2.910		3.041		3.070		3.136	
No-CP	1.883		2.285		2.473		2.571	
FPG, mmol/L		0.744		<0.001		<0.001		0.100
CP	5.303		5.887		6.179		6.308	
No-CP	5.258		5.503		5.878		6.112	
LDL-C/HDL-C ratio		0.057		<0.001		<0.001		<0.001
CP	1.661		2.129		2.443		2.511	
No-CP	1.191		1.539		1.842		2.059	

SBP, systolic blood pressure; DBP, diastolic blood pressure;; FPG, fasting plasma glucose; TC, total cholesterol; TG, triglycerides; HDL-C, high density lipoprotein cholesterol; LDL-C, low density lipoprotein cholesterol

Waist circumference, TC, and FPG were significantly higher in patients with carotid plaque in the normal-weight and overweight groups (all *P* < 0.01). SBP was higher in patients with carotid plaque for all groups except the underweight group (*P* = 0.509). However, DBP was only higher in patients with carotid plaque in the overweight group (*P* = 0.001). HDL-C was lower in patients with carotid plaque in the overweight group (*P* = 0.022), while LDL-C was higher in patients with carotid plaque in all four groups (all *P* < 0.01). Furthermore, similar to SBP, the LDL-C/HDL-C ratio was also higher in patients with carotid plaque in the normal-weight, overweight, and obese groups (all *P* < 0.001).

### Multiple linear regression analysis of conventional risk factors associated with carotid plaque

A multivariate analysis was performed to assess the relationships between carotid plaque and risk factors by BMI. Age and LDL-C were independent risk factors for carotid plaque presence in the underweight group. The risk of carotid plaque was increased by at least 10.59-fold with older age and by 2.60-fold for each 1-unit increase in LDL-C (all *P* < 0.05). Age, SBP, FPG, and LDL-C were positively associated with carotid plaque in the normal-weight group. The risk of carotid plaque increased by at least 1.18-fold with older age and by 61% for each 1-unit increase in LDL-C (all *P* < 0.001). SBP and FPG showed a slight association with carotid plaque (OR, 1.01 and 1.11; *P* = 0.005 and 0.046, respectively). Male sex, older age, SBP, and LDL-C were statistically significantly correlated with carotid plaque presence in the overweight and obese groups (all *P* < 0.05). SBP also showed a weak association with carotid plaque in the overweight and obese groups (OR, 1.02 and 1.01, respectively). Moreover, the significant relationship between education level, smoking, waist circumference, DBP, and the LDL-C/HDL-C ratio disappeared following adjustment for other confounding factors (all *P* > 0.05) (Table 6).

**Table 6:** Adjusted OR (95% CI) of risk factors association with carotid plaque by BMI group

Risk factors	References	Underweight		Normal		Overweight		Obesity	
		OR (95% CI)	*P*	OR (95% CI)	*P*	OR (95% CI)	*P*	OR (95% CI)	*P*
Men	Women	2.68 (0.75, 9.56)	0.130	1.37 (0.99, 1.91)	0.058	1.97 (1.45, 2.68)	<0.001	1.77 (1.18, 2.66)	0.006
Age group	45∼54 years								
55∼64 years		12.73 (1.49, 109.09)	0.020	2.18 (1.53, 3.10)	<0.001	2.67 (1.98, 3.60)	<0.001	2.18 (1.53, 3.10)	<0.001
65∼74 years		18.99 (1.99, 181.13)	0.011	2.95 (1.92, 4.54)	<0.001	3.17 (2.18, 4.63)	<0.001	2.34 (1.45, 3.76)	<0.001
≥75 years		11.59 (1.17, 115.00)	0.036	5.42 (3.12, 9.41)	<0.001	7.24 (4.13, 12.68)	<0.001	4.35 (2.01, 9.43)	<0.001
Education group	≥6 years								
0 years		—	—	0.90 (0.58, 1.39)	0.630	0.90 (0.62, 1.31)	0.593	1.16 (0.71, 1.87)	0.561
1∼6 years		—	—	1.01 (0.74, 1.39)	0.944	0.98 (0.75, 1.28)	0.874	1.21 (0.85, 1.72)	0.282
Smoking	Never smoking	—	—	1.37 (0.97, 1.94)	0.076	1.20 (0.86, 1.67)	0.274	0.93 (0.60, 1.45)	0.753
WC	—	—	—	1.01 (0.99, 1.03)	0.336	0.99 (0.97, 1.01))	0.447	—	—
SBP	—	—	—	1.01 (1.00, 1.02)	0.005	1.02 (1.01, 1.03)	<0.001	1.01 (1.00, 1.02)	0.004
DBP		—	—	—	—	0.99 (0.98, 1.00)	0.156	—	—
FPG	—	—	—	1.11 (1.00, 1.23)	0.046	1.06 (0.99, 1.14)	0.092	—	—
HDL-C		—	—	—	—	0.81 (0.61, 1.07)	0.141	—	—
LDL-C	—	3.60 (1.34, 9.72)	0.011	1.61 (1.32, 1.97)	<0.001	1.61 (1.45, 1.79)	<0.001	1.41 (1.14, 1.74)	0.001
LDL-C/HDL-C	—	—	—	1.10 (0.87, 1.40)	0.414	—	—	1.07 (0.84, 1.36)	0.612

SBP, systolic blood pressure; DBP, diastolic blood pressure;; FPG, fasting plasma glucose; HDL-C, high density lipoprotein cholesterol; LDL-C, low density lipoprotein cholesterol

## DISCUSSION

This is the first report to describe the associations between BMI and carotid plaque in a low-income population with a high incidence of stroke in rural China. Significant correlations were observed between the presence of carotid plaque and male sex, older age, SBP, FPG, and LDL-C among the four BMI subgroups. Of those factors, a positive association was found between carotid plaque and both older age and LDL-C among all four subgroups. Male sex increased the risk of carotid plaque in the overweight and obese groups. SBP was an independent risk factor for carotid plaque in the normal-weight, overweight, and obese groups. Moreover, FPG was only significantly positively correlated with carotid plaque in the normal-weight group. No significant links between carotid plaque and education level, cigarette smoking, waist circumference, DBP, HDL-C, or the LDL-C/HDL-C ratio were observed in this study.

Several studies demonstrated that age is a strong risk factor for carotid plaque [6, 9, 10, 22]. In a traditional vascular risk factor model, Kuo et al. [6] demonstrated that older age was significantly associated with carotid plaque presence (B = 0.332, *P* < 0.0001). A community-based study from Iceland [9] explored the prevalence and determinants of carotid plaque in 6524 participants aged 25-69 years. A multinomial logistic regression analysis in that study showed that older age was a risk factor for carotid plaque. The risk of carotid plaque increased by 0.97-fold for each 5-year increase in age (95% CI, 1.83-2.12, *P* < 0.001). Our study also showed that older age was a strong risk factor for CP. Older age was significantly correlated with carotid plaque among all four BMI subgroups. The risk of carotid plaque presence increased by at least 1.18-fold for older individuals compared to that in younger individuals.

The relationship between serum lipid profiles and carotid plaque has been widely studied worldwide. However, there are conflicting results with respect to the relationship between LDL-C and carotid plaque. Mi et al. [23] reported that LDL-C had no significant association with CP. They recruited 22,222 urban or rural residents who were at high risk of stroke and not using any drugs that might affect serum lipids. After controlling for age, sex, education, traditional risk factors, history of stroke, and family history of stroke, a multivariate analysis showed no association between carotid plaque and LDL-C (OR, 0.976; 95% CI, 0.914-1.043; *P* = 0.475). In contrast to those results, Yang et al. [19] concluded that LDL-C was a risk factor for carotid plaque presence (OR, 1.325; 95% CI, 1.046-1.821; *P* = 0.033) in a multivariate model in which the presence of carotid plaque was the dependent variable and age, LDL-C, and HDL-C were the independent variables. The results of the present study showed that LDL-C was a strong risk factor for carotid plaque among the four weight groups. The risk of carotid plaque developing increased by ≥41% for each 1-mmol/L increase in the serum concentration of LDL-C. The underlying mechanism might be explained by oxidized LDL-C, which could enter and accumulate within the arterial walls and be involved in the inflammatory process in atherosclerosis [24].

Several studies have shown that male sex is a risk factor for carotid plaque [6, 9]. In a traditional vascular risk factor model, male sex contributed to carotid plaque development (B = 0.084, *P* < 0.002) [6]. Sturlaugsdottir et al. [9] reported that male sex was a risk factor for CP (OR, 1.53; 95% CI, 1.22-1.92, *P* < 0.001). The results of the present study were consistent with those results. Male sex was a risk factor for carotid plaque among both the overweight and obese groups. However, the significant relationship between male sex and carotid plaque among the underweight and normal-weight groups disappeared in the multivariate model, which may be explained by the observation that the numbers of carotid plaque patients in the underweight and normal-weight groups were too small to reach statistical significance.

Gardener et al. [6] and Sturlaugsdottir et al. [9] also showed that SBP significantly affected the development of carotid plaque. In the study by Gardener et al., SBP positively increased the risk of carotid plaque (B = 0.159, *P* < 0.0001). Sturlaugsdottir et al. revealed that each 10-mmHg increase in SBP increased the risk of carotid plaque by 20% (95% CI, 1.15-1.26; *P* < 0.001). Similar to their research, our study also showed that SBP was a risk factor for carotid plaque in the normal-weight, overweight, and obese groups. However, the effect of SBP on carotid plaque development was limited; the risk of carotid plaque increased by ≤2% for each 1-unit increase in SBP. Furthermore, Yin et al. [25] conducted a population-based study of 4,992 individuals in Hang Zhou, China, and they reported that FPG was a risk factor for carotid plaque in both men and women (OR, 1.49; 95% CI, 1.21-1.84, *P* < 0.0001 and OR, 1.93; 95% CI, 1.31-2.84, *P* = 0.001, respectively). Consistent with Yin et al.'s report, the present study showed that FPG was a risk factor for carotid plaque in the normal-weight group, although FPG only slightly increased the risk for carotid plaque (by 11%).

In addition, although previous studies have shown that education level [6], cigarette smoking [6, 9, 10, 12–14], DBP [26, 27], HDL-C [10, 19], and the LDL-C/HDL-C ratio [10, 19] were predictors of carotid plaque, no significant link between carotid plaque and education level, cigarette smoking, DBP, HDL-C, or the LDL-C/HDL-C ratio was observed in this study. This finding might be explained by the fact that the previous study did not analyze the association between those variables with carotid plaque after stratification by BMI, which could have led to the disappearance of statistical significance.

This study has some limitations. (1) An inherent limitation of our cross-sectional study design is that it cannot determine the causal correlation between carotid plaque and the variables found to be significant; thus, further longitudinal studies are warranted to determine causality. (2) The study population consisted of low-income adults ≥45 years living in a rural area of northern China, so the conclusions of the present study may not be generalizable to other populations. However, considering that low-income, rural residents comprise > 50% of the total population of China, the results of the present study are important for further study. (3) We did not collect information regarding medication use for all subjects; however, due to the participants’ low socioeconomic status, the frequency of medication use was lower in this population, so it is likely that it did not affect the validity of the present results.

This is the first population-based study to explore the association between BMI and carotid plaque presence in rural China. All participants were low-income residents with low educational attainment aged 45 years and older. To our knowledge, no similar report has revealed an association between BMI and carotid plaque. The multivariate analysis showed that older age and LDL-C were risk factors for carotid plaque among all four BMI subgroups. Male sex was a risk factor for carotid plaque presence in the overweight and obese groups. SBP was an independent risk factor for carotid plaque in the normal-weight, overweight, and obese groups. FPG was a risk factor for carotid plaque in the normal-weight group.

LDL-C was a strong independent risk factor for carotid plaque among all groups. Therefore, the findings of this study suggest that it is necessary to control LDL-C in this population. In addition, controlling SBP and FPG will also assist in reducing the carotid plaque risk. Furthermore, our study provided specific information for clinicians regarding specific preventive strategies for different populations by BMI.

## MATERIALS AND METHODS

### Study population

The present study was a population-based, cross-sectional study conducted from April 2014 to January 2015. The enrolled population was from the Tianjin Brain Study [28–31]. In brief, the total population comprised 14,251 participants from among 18 administrative villages in rural Tianjin, China. About 95% of participants were low-income farmers, with an average disposable annual income < $1600 US in 2014 [32]. Residents aged ≥45 years without CVDs were included in this study, while those with a previous history of CVD were excluded.

All investigative protocols were approved by the ethics committee of Tianjin Medical University General Hospital; the methods were carried out in accordance with the approved guidelines, and informed consent was obtained from all participants.

### Information collection and risk factor definitions

All variables in this study were obtained by trained epidemiological researchers through face-to-face interviews. A pre-specified questionnaire was used to collect all information for this study.

Demographic information, including name, sex, date of birth, and educational level, were derived from previous records. All participants were separated into four age groups: 45-54 years, 55-64 years, 65-74 years, and ≥75 years. Educational level was classified into three groups according to the length of the individuals’ formal education: illiteracy (without a formal education), 1-6 years group, and > 6 years group.

Previous individual and family medical histories, including of hypertension, DM, stroke, transient ischemic attack, and coronary heart disease, were obtained according to patient self-report or previous records.

Lifestyle characteristics of interest included cigarette smoking and alcohol consumption. Cigarette smoking was defined as smoking more than 1 cigarette per day for at least 1 year, and participants were categorized as never smokers and smokers. Alcohol consumption was defined as drinking more than 500 grams of alcohol per week for at least 1 year, and participants were divided into the never alcohol consumption group and alcohol consumption group.

### Physical examination

Measurements of blood pressure (including SBP and DBP), height, and weight were measured in the local village clinic during the baseline survey; levels of FPG, TC, TG, HDL-C, and LDL-C in the serum were assessed at the Ji County People's Hospital. Carotid ultrasonography and 12-lead echocardiography were also conducted by a professional. BMI was calculated as the participant's weight (kg) divided by the square of height (m^2^).

Hypertension was defined as SBP ≥140 mmHg, DBP ≥90 mmHg, or taking medication for hypertension. DM was defined as FPG ≥7.0 mmol/L or taking insulin or oral hypoglycemic medications.

### Ultrasonography measurements

One trained technician blinded to individuals’ previous disease histories performed all ultrasound examinations. The patients were examined when they were in the supine position using B-mode ultrasonography (Terason 3000; Burlington, MA, US) with a 5-12-MHz linear array transducer. Plaques are focal structures that encroach into the arterial lumen by at least 0.5 mm or 50% of the surrounding intima-media thickness, or demonstrate a thickness of > 1.5 mm, as measured from the intima-lumen interface to the media-adventitia interface [33]. Subjects with carotid plaque were defined as having at least one lesion, not based on the total number of carotid plaques.

### Statistical analyses

Continuous variables were presented as means with standard deviations, and Student's *t*-test or an analysis of variance was used to compare differences between two groups or multiple groups, respectively. Categorical variables were presented as numbers with frequencies and were compared using the chi-squared test. Multiple linear regression analyses were used to evaluate the associations between traditional risk factors and the presence of carotid plaques. We performed multivariate logistic regression analyses to evaluate the determinants of carotid plaque after adjusting for other confounding factors. The results are presented as adjusted ORs and 95% CIs. *P* values < 0.05 in two-tailed tests were considered statistically significant. SPSS for Windows (version 19.0; SPSS Inc., Chicago, IL, USA) was used for analyses.

## Abbreviations

BMI: body mass index
CI: confidence interval
CVD: cardiovascular disease
DBP: diastolic blood pressure
DM: diabetes mellitus
FPG: fasting plasma glucose
HDL-C: high-density lipoprotein cholesterol
LDL-C: low-density lipoprotein cholesterol
OR: odds ratio
TC: total cholesterol
TG: triglycerides
SBP: systolic blood pressure
